# Factors associated with women’s childbirth experience in Slovakia: A cross-sectional analysis of overall childbirth experience and individual domains of the Childbirth Experience Questionnaire 2 (CEQ2)

**DOI:** 10.18332/ejm/226841

**Published:** 2026-09-09

**Authors:** Martina Bašková, Iveta Slotová, Zuzana Škodová, Erika Maskálová

**Affiliations:** 1 Jessenius Faculty of Medicine, Department of Midwifery, Comenius University, Martin, Slovakia

**Keywords:** childbirth experience, childbirth experience questionnaire 2, woman-centered care, parity, mode of birth

## Abstract

**INTRODUCTION:**

The experience of a woman with childbirth represents a significant indicator of the quality of maternal care and influences their health during the postpartum period. The aim of this cross-sectional study was to analyze women's childbirth experiences in Slovakia using the Childbirth Experience Questionnaire 2 (CEQ2) and to identify associated demographic and obstetric factors.

**METHODS:**

Using a consecutive sampling strategy, all women giving birth in the participating hospital between September 2024 and March 2025 who met the eligibility criteria were included. The final sample was 103 women. Four CEQ2 domains (own capacity, perceived safety, professional support, participation) were assessed as well as the overall CEQ2 score. Data were analyzed using multiple linear regression.

**RESULTS:**

The mean overall CEQ2 score was 2.74 (SD=0.37). Among the domains, participation received the highest mean score (3.00 ± 0.59), while own capacity received the lowest (2.57 ± 0.52). Multiparity was associated with a higher overall CEQ2 score (B=5.66; 95% CI: 2.41–8.91; p<0.001) and with higher scores in the domains own capacity and perceived safety. Operative vaginal delivery was associated with a lower overall CEQ2 score (B= -6.06; 95% CI: -11.59 – -0.53; p=0.032) and lower perceived safety scores. No demographic or obstetric factors were associated with the professional support or participation domains.

**CONCLUSIONS:**

Women generally reported positive childbirth experiences. Multiparity and operative vaginal birth showed the most consistent associations with the overall CEQ2 score. These findings should be confirmed in larger multi-center and longitudinal studies.

## INTRODUCTION

A positive childbirth experience is increasingly recognized as a fundamental indicator of high-quality maternity care and an essential component of woman-centered healthcare^1,2^. Contemporary maternity services are expected not only to achieve favorable maternal and neonatal clinical outcomes, but also to provide respectful, safe and emotionally supportive care that responds to women’s individual needs, values and preferences^3,4^. In its recommendations on intrapartum care, the World Health Organization stresses that every woman has the right to a positive childbirth experience characterized by respectful care, effective communication, informed decision-making, and continuous support throughout labor and birth^2^. Consequently, women’s experiences of childbirth have become an important patient-reported outcome and a key measure of quality in maternity care^1,3^.

The childbirth experience has important short- and long-term consequences for maternal and child health. Positive experiences have been associated with improved psychological well-being, greater maternal self-efficacy, stronger mother–infant bonding, successful breastfeeding, and greater confidence in future pregnancies^5,6^. On the contrary, negative childbirth experiences increase the risk of postpartum depression, posttraumatic stress symptoms, fear of childbirth, impaired maternal-infant attachment, and avoidance of subsequent pregnancy^7,8^. These findings highlight that women’s subjective perceptions of childbirth are not merely reflections of clinical outcomes, but represent an important determinant of maternal health and well-being. Current evidence indicates that childbirth experience is a multidimensional construct resulting from the interaction of obstetric, psychological, interpersonal, and organizational factors^9,10^. Among clinical determinants, parity and mode of birth are the most consistently associated with childbirth experience, with multiparous women and those with spontaneous vaginal birth generally reporting more positive experiences^11-13^. However, evidence on other potential determinants, including labor interventions, birth-related trauma, pain management, and prebirth expectations, remains inconsistent, with studies reporting conflicting associations with childbirth experience^5,8,14,15^. This heterogeneity suggests that the childbirth experience is shaped not only by objective clinical circumstances but also by their subjective interpretation and that the relative contribution of individual determinants may vary across populations and healthcare settings. Valid assessment of childbirth experience requires reliable and culturally appropriate measurement instruments. The Childbirth Experience Questionnaire 2 (CEQ2) is one of the most widely used patient-reported outcome measures for evaluating women’s experiences during labor and birth^10^. The Childbirth Experience Questionnaire was originally developed and validated in Sweden as a multidimensional measure of women’s experiences of labor and birth. The revised version, CEQ2, was subsequently developed to strengthen the assessment of women’s participation in decision-making and aspects of professional support^10^. Since its development, the CEQ2 has been translated and psychometrically validated in several countries and has become the internationally accepted instrument for comparing women’s childbirth experiences across different maternity care systems^16-18^.

Despite the growing body of international evidence, important geographical disparities remain. Most studies evaluating childbirth experience using CEQ2 have been conducted in Northern and Western Europe or other high-income countries where woman-centered maternity care is well established^17-19^. In contrast, evidence from Central and Eastern Europe remains limited. Differences in healthcare organization, professional autonomy of midwives, implementation of respectful maternity care, and women’s participation in clinical decision-making can substantially influence childbirth experiences and limit the direct applicability of findings from other healthcare settings^19^.

In Slovakia, research that evaluates childbirth experiences using internationally validated instruments is still scarce. Although maternal care has undergone gradual improvements during recent decades, childbirth continues to be provided predominantly within a hospital-based obstetric model, and the implementation of woman-centered care may vary between maternity hospitals. Evidence regarding factors associated with women’s childbirth experiences is therefore needed to support quality improvement initiatives, strengthen respectful maternity care, and inform evidence-based maternity policies. Understanding women’s childbirth experiences is therefore essential for identifying areas where maternity care can be further improved^20-22^. In Slovakia, women’s childbirth experiences using the CEQ/CEQ2 have been evaluated in only two previous studies. Maskálová et al.^21^ used a translated but non-validated version of the original CEQ in a sample of primiparous women, while Henriksen et al.^17^ adapted and validated a Slovak version (CEQ-SK) based on a combination of the CEQ and CEQ2, which revealed a three-factor rather than the original four-domain structure. To our knowledge, the present study is the first to apply the internationally validated four-domain structure of the CEQ2 in a Slovak sample of both primiparous and multiparous women, thus providing evidence directly comparable with international CEQ2 studies using the original domain structure.

This study aimed to evaluate childbirth experience among postpartum women in Slovakia using a Slovak translation of the internationally validated Childbirth Experience Questionnaire 2 (CEQ2) and to identify demographic and obstetric factors associated with the overall childbirth experience and its individual domains. By providing evidence from an under-represented Central European setting, this study contributes to the international literature on childbirth experience and offers context-specific evidence to support further improvements in woman-centered maternal care.

## METHODS

### Study design

This cross-sectional study was conducted to evaluate women’s childbirth experiences and identify demographic and obstetric factors associated with overall childbirth experience and its individual domains. The study report followed the Strengthening the Reporting of Observational Studies in Epidemiology (STROBE) guidelines. Data were collected once during the early postpartum period. Women were approached on the second or third postpartum day while hospitalized on the postnatal ward, allowing immediate assessment of childbirth experiences after birth and minimizing recall bias.

### Setting and participants

The study was carried out in a public regional maternity hospital in western Slovakia that serves a population of approximately 300000 inhabitants. Women were recruited consecutively between September 2024 and March 2025 during their stay in the postnatal ward. All women who delivered within the given period and met eligibility criteria were included.

Eligibility criteria included having given birth vaginally or by cesarean section, having delivered a healthy newborn without serious neonatal complications, being able to read and understand the Slovak language, and providing written informed consent to participate in the study. Women whose newborn required intensive neonatal care or who were unable to complete the questionnaire independently were excluded.

### Data collection

**Figure 1:**
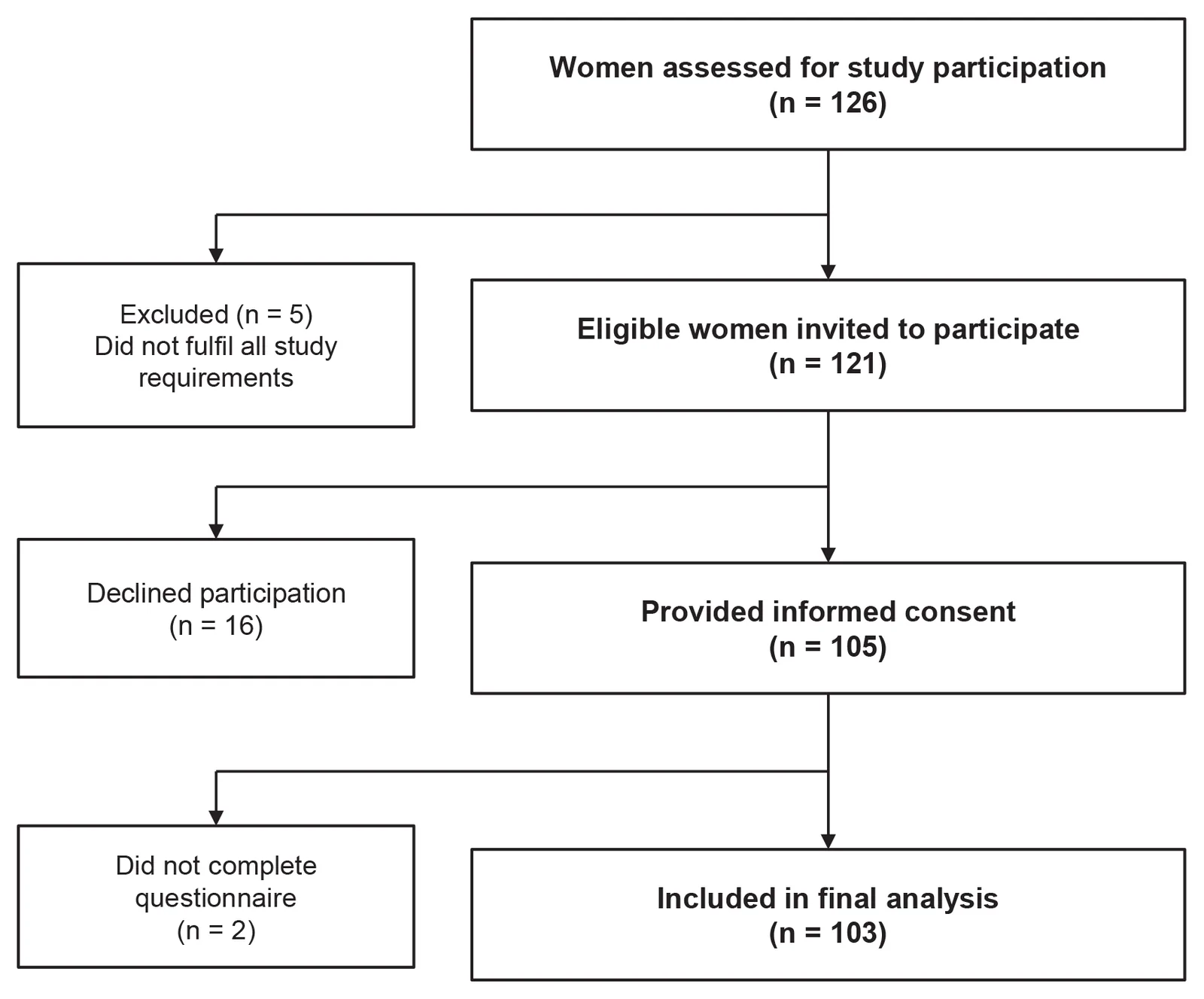
Flow diagram of participant recruitment and inclusion women assessed for study participation

Potential participants were approached by trained midwives who explained the purpose of the study and invited eligible women to participate. Participation was voluntary and anonymous, and no financial or other incentives were offered. The women completed the questionnaire independently after receiving written and verbal information about the study. Completed questionnaires were returned in sealed envelopes to ensure confidentiality. A total of 126 women were assessed for participation in the study. Of these, 5 women did not meet all study requirements and were therefore not enrolled. The remaining 121 eligible women were invited to participate, of whom 16 declined participation. A total of 105 women gave their written informed consent; however, two women did not complete the questionnaire. Consequently, data from 103 women were included in the final analysis, corresponding to a response rate of 85.1% among eligible invited women (Figure 1).

### Study instrument

Childbirth experience was assessed using the Childbirth Experience Questionnaire 2 (CEQ2), following permission from the original instrument developers^10^. The CEQ2 is a validated patient-reported outcome measure designed to evaluate women’s experiences during labor and delivery in four domains: own capacity, perceived safety, professional support, and participation. The questionnaire comprises 22 items. Nineteen items are rated on a 4-point Likert scale, whereas three items use a 100 mm visual analog scale (VAS). VAS responses were transformed into four response categories according to the original scoring instructions (0–40=1; 41–60=2; 61–80=3; 81–100=4). Negatively worded items were reverse-coded so that higher scores consistently indicated more positive childbirth experiences. Domain scores and the overall CEQ2 score were calculated as the arithmetic mean of the corresponding items, with higher scores indicating a more positive childbirth experience.

For the purposes of this study, the original English version of the CEQ2 was translated into Slovak using a forward-backward translation procedure performed by bilingual experts to ensure conceptual and linguistic equivalence with the original instrument. The original wording, scoring system, and four-domain structure were retained without modification. No formal pilot tests or psychometric validation of the Slovak translation was performed before its implementation, as the objective of the present study was to investigate demographic and obstetric factors associated with childbirth experience using a Slovak translation of the original CEQ2 rather than to validate the instrument. The internal consistency of the overall CEQ2 in the present study was acceptable (Cronbach’s α=0.760). Cronbach’s alpha coefficients for the individual domains were 0.709 for own capacity, 0.619 for perceived safety, 0.564 for professional support, and 0.446 for participation.

The questionnaire was supplemented with demographic and obstetric variables that included maternal age, education level, parity, mode of birth, and birth-related injury.

### Statistical analysis

Data were analyzed using Jamovi (version 2.6.26) and Microsoft Excel 365. Descriptive statistics were used to summarize the characteristics of the participants and the CEQ2 scores. Continuous variables are presented as means and standard deviations (SDs), whereas categorical variables are reported as frequencies and percentages. The descriptive results of CEQ2 are presented as mean scores (range: 1–4) to facilitate comparison with previous CEQ2 studies. As no missing data were identified, all analyses were performed using complete case data and no imputation procedures were required.

Separate multiple linear regression analyses were performed to examine demographic and obstetric factors associated with the overall CEQ2 total score and the total scores of each of its four domains. Independent variables included maternal age, education level, parity, mode of birth, and birth-related injury. Regression results are presented as unstandardized regression coefficients (B), 95% confidence intervals (95% CI), and p-values. Statistical significance was set at p<0.05.

Maternal age, education level, parity, mode of birth, and birth-related injury were selected *a priori* as adjustment variables based on their established or hypothesized associations with childbirth experience in previous CEQ/CEQ2 studies^11-15^. Maternal age was entered as a continuous variable (years). The education level was coded as a binary variable (primary/secondary education vs university education), with primary/secondary education serving as the reference category. Parity was coded as primiparous or multiparous, with primiparity as a reference category. The mode of birth was entered as a variable of three categories (spontaneous vaginal birth, cesarean section, and operative vaginal birth), with spontaneous vaginal birth as the reference category. Birth-related injury was entered as a three-category variable (no injury, episiotomy, and perineal rupture), with no injury as the reference category. Categorical variables were entered using indicator coding, and all independent variables were entered simultaneously into each model (forced entry method).

Multicollinearity was assessed using variance inflation factors (VIF) and tolerance statistics. No evidence of problematic multicollinearity was identified (VIF: 1.10–1.21; tolerance: 0.823–0.913).

### Ethical considerations

The study was approved by the Ethics Committee of the Faculty Hospital Trnava, Slovakia (No. R-2/16082024/ETK, 16 August 2024) and was carried out in accordance with the Declaration of Helsinki^23^. Written informed consent was obtained from all participants before enrolment.

## RESULTS

### Characteristics of the participants

Table 1 summarizes the sociodemographic and obstetric characteristics of the study participants. A total of 103 women were included in the study. Most of the participants were aged 30–34 years (37.9%), had university education (60.2%) and were multiparous (50.5%). Spontaneous vaginal delivery was the most common mode of birth (61.2%), followed by cesarean section (30.1%) and operative vaginal delivery (8.7%). Episiotomy was the most common birth injury (50.5%), while 32.0% of women did not experience birth injury and 17.5% sustained a perineal rupture.

**Table 1:** Sociodemographic and obstetric characteristics of women at a public regional maternity hospital in western Slovakia, September 2024–March 2025 (N=103)

*Characteristics*	*n (%)*
**Age** (years)	
<25	14 (13.6)
25–29	33 (32.0)
30–34	39 (37.9)
≥35	17 (16.5)
**Education level**	
Primary/secondary	41 (39.8)
University	62 (60.2)
**Parity**	
Primiparous	51 (49.5)
Multiparous	52 (50.5)
**Mode of birth**	
Spontaneous vaginal delivery	63 (61.2)
Cesarean section	31 (30.1)
Operative vaginal delivery	9 (8.7)
**Birth-related injury**	
None	33 (32.0)
Episiotomy	52 (50.5)
Perineal rupture	18 (17.5)

### Overall childbirth experience

Table 2 presents the overall CEQ2 score and the scores of the four individual domains. The mean overall CEQ2 score was 2.74 (SD=0.37). Among the four domains, participation achieved the highest mean score (3.00 ± 0.59), followed by perceived safety (2.86 ± 0.55), professional support (2.69 ± 0.59), and own capacity (2.57 ± 0.52).

**Table 2:** Overall and domain-specific Childbirth Experience Questionnaire 2 (CEQ2) scores, at a public regional maternity hospital in western Slovakia September 2024–March 2025 (N=103)

*Outcome*	*Mean (SD)*
**Overall CEQ2 score**	2.74 (0.37)
**Own capacity**	2.57 (0.52)
**Perceived safety**	2.86 (0.55)
**Professional support**	2.69 (0.59)
**Participation**	3.00 (0.59)

SD: standard deviation. Higher scores indicate a more positive childbirth experience.

Table 3 presents the mean overall CEQ2 score and domain-specific scores according to participants’ sociodemographic and obstetric characteristics. Multiparous women had higher mean CEQ2 scores across all domains than primiparous women. The highest mean overall CEQ2 score was observed among women who experienced spontaneous vaginal delivery (2.81 ± 0.33), while the lowest was observed among those who underwent operative vaginal delivery (2.48 ± 0.38). The differences according to maternal age, education level, and birth-related injury were small.

**Table 3:** Overall Childbirth Experience Questionnaire 2 (CEQ2) score and domain-specific scores according to participants’ sociodemographic and obstetric characteristics, at a public regional maternity hospital in western Slovakia September 2024–March 2025 (N=103)

*Characteristics*	*n (%)*	*Own capacity Mean (SD)*	*Perceived safety Mean (SD)*	*Professional support Mean (SD)*	*Participation Mean(SD)*	*Overall CEQ2 score Mean (SD)*
**Age** (years)						
<25	14 (13.6)	2.20 (0.51)	2.71 (0.53)	2.61 (0.69)	2.93 (0.52)	2.53 (0.35)
25–29	33 (32.0)	2.67 (0.52)	2.88 (0.58)	2.65 (0.71)	3.01 (0.77)	2.77 (0.39)
30–34	39 (37.9)	2.55 (0.53)	2.81 (0.57)	2.69 (0.51)	2.97 (0.52)	2.71 (0.38)
≥35	17 (16.5)	2.72 (0.34)	3.06 (0.40)	2.85 (0.40)	3.10 (0.38)	2.89 (0.22)
**Education level**						
Primary/secondary	41 (39.8)	2.53 (0.55)	2.92 (0.48)	2.76 (0.56)	2.75 (0.34)	2.66 (0.23)
University	62 (60.2)	2.60 (0.50)	2.83 (0.59)	2.65 (0.61)	2.73 (0.39)	2.68 (0.26)
**Parity**						
Primiparous	51 (49.5)	2.33 (0.50)	2.69 (0.51)	2.64 (0.59)	2.92 (0.51)	2.58 (0.31)
Multiparous	52 (50.5)	2.80 (0.43)	3.04 (0.53)	2.74 (0.59)	3.08 (0.66)	2.89 (0.36)
**Mode of birth**						
Spontaneous vaginal delivery	63 (61.2)	2.64 (0.48)	2.93 (0.51)	2.83 (0.46)	3.03 (0.68)	2.81 (0.33)
Cesarean section	31 (30.1)	2.52 (0.58)	2.89 (0.55)	2.42 (0.69)	2.89 (0.43)	2.65 (0.40)
Operative vaginal delivery	9 (8.7)	2.26 (0.48)	2.31 (0.54)	2.64 (0.74)	3.15 (0.29)	2.48 (0.38)
**Birth-related injury**						
None	33 (32.0)	2.62 (0.54)	2.90 (0.58)	2.55 (0.68)	2.92 (0.66)	2.72 (0.42)
Episiotomy	52 (50.5)	2.48 (0.50)	2.82 (0.47)	2.84 (0.42)	3.11 (0.90)	2.74 (0.28)
Perineal rupture	18 (17.5)	2.58 (0.47)	2.82 (0.60)	2.84 (0.49)	3.04 (0.48)	2.77 (0.36)

SD: standard deviation. Descriptive statistics only, no statistical test was performed.

### Factors associated with childbirth experience

Separate multiple linear regression models were performed for the overall CEQ2 total score and the total scores for the four CEQ2 domains (Table 4). Multicollinearity diagnostics indicated no evidence of problematic multicollinearity among predictors. The VIF values ranged from 1.10 to 1.21, and the tolerance values ranged from 0.823 to 0.913.

**Table 4:** Multiple linear regression analyses examining demographic and obstetric factors associated with the overall CEQ2 score and individual CEQ2 domains, at a public regional maternity hospital in western Slovakia, September 2024–March 2025 (N=103)

*Factor*	*Overall CEQ2 total score B (95% CI), p*	*Own capacity B (95% CI), p*	*Perceived safety B (95% CI), p*	*Professional support B (95% CI), p*	*Participation B (95% CI), p*
Age (years)	0.25 (-0.08–0.57), 0.133	0.12 (-0.05–0.28), 0.166	0.07 (-0.07–0.20), 0.323	0.07 (-0.05–0.20), 0.244	-0.01 (-0.09–0.07), 0.785
University vs primary/secondary education	0.00 (-3.23–3.23), 0.999	-0.98 (-2.61–0.66), 0.239	0.25 (-1.12–1.61), 0.720	0.88 (-0.37–2.13), 0.165	-0.15 (-0.94–0.63), 0.702
Multiparous vs primiparous	**5.66 (2.41–8.91), <0.001**	**3.00 (1.35–4.64), <0.001**	**1.70 (0.33–3.08), 0.016**	0.28 (-0.98–1.54), 0.656	0.68 (-0.12–1.47), 0.094
Cesarean section vs spontaneous vaginal delivery	-2.55 (-6.96–1.87), 0.255	-1.43 (-3.66–0.81), 0.208	0.24 (-1.62–2.11), 0.797	-1.43 (-3.14–0.28), 0.100	0.07 (-1.01–1.14), 0.902
Operative vaginal delivery vs spontaneous vaginal delivery	**-6.06 (-11.59 – -0.53), 0.032**	-2.45 (-5.25–0.34), 0.085	**-3.21 (-5.55 – -0.88), 0.008**	-0.82 (-2.97–1.32), 0.448	0.43 (-0.92–1.78), 0.527
Episiotomy vs no birth injury	-0.90 (-5.32–3.52), 0.688	1.12 (-1.12–3.35), 0.323	-0.45 (-2.32–1.42), 0.632	-0.86 (-2.57–0.86), 0.323	-0.71 (-1.79–0.37), 0.195
Perineal rupture vs no birth injury	-0.22 (-4.56–4.13), 0.922	0.11 (-2.09–2.31), 0.920	-0.13 (-1.97–1.70), 0.886	0.15 (-1.53–1.84), 0.859	-0.35 (-1.40–0.71), 0.519
Adjusted R²	**0.188**	**0.200**	**0.118**	**0.074**	**-0.014**
Model p-value	**<0.001**	**<0.001**	**0.008**	**0.045**	**0.585**

B: unstandardized regression coefficient. CI: confidence interval. CEQ2: Childbirth Experience Questionnaire 2. Regression models were adjusted for maternal age, education level, parity, mode of birth and birth-related injury. Maternal age was entered as a continuous variable. Reference categories were primary/secondary education, primiparity, spontaneous vaginal delivery and no birth injury. Bold values indicate statistical significance at p<0.05.

The regression model for the overall CEQ2 total score was statistically significant (adjusted R²=0.188, p<0.001). Multiparity was independently associated with a higher overall CEQ2 total score (B=5.66; 95% CI: 2.41–8.91, p<0.001), whereas operative vaginal delivery was associated with a lower overall CEQ2 total score (B= -6.06; 95% CI: -11.59 – -0.53, p=0.032).

For the own capacity domain, multiparity was the only factor independently associated with higher scores (B=3.00; 95% CI: 1.35–4.64, p<0.001; adjusted R²=0.200). In the perceived safety domain, multiparity was associated with higher scores (B=1.70; 95% CI: 0.33–3.08, p=0.016), while operative vaginal delivery was associated with lower scores (B= -3.21; 95% CI: -5.55 – -0.88, p=0.008; adjusted R²=0.118). No demographic or obstetric factors were independently associated with the professional support or participation domains after adjustment for potential confounders. Although the overall model for professional support was statistically significant (adjusted R²=0.074, p=0.045), no individual factor reached statistical significance. The regression model for participation was not statistically significant (adjusted R²= -0.014, p=0.585) (Table 4).

## DISCUSSION

The present study provides one of the first comprehensive evaluations of childbirth experience among postpartum women in Slovakia using a Slovak translation of the internationally validated Childbirth Experience Questionnaire 2 (CEQ2). In general, women reported a mostly positive childbirth experience, although important differences were observed between the individual dimensions of the questionnaire. Among the variables examined, parity emerged as the most consistent factor associated with the childbirth experience. Multiparity was independently associated with higher overall CEQ2 scores and more favorable ratings in the own capacity and perceived safety domains. In addition, operative vaginal delivery was associated with a less overall positive experience of childbirth and a lower perceived safety. Collectively, these findings reinforce the growing body of evidence indicating that childbirth experience is a multidimensional construct influenced by both obstetric factors and the organization of maternity care^9,10^.

One of the most important findings of the present study was the consistently positive association of multiparity with the women’s childbirth experience. Multiparous women reported significantly higher overall CEQ2 scores and achieved better ratings in the own capacity and perceived safety domains than primiparous women. Similar findings have been consistently reported in previous studies conducted in Sweden, Iceland, Spain, and the United Kingdom, where previous childbirth experience was identified as one of the strongest predictors of maternal satisfaction^11-13^. Although the relationship between parity and childbirth experience has been repeatedly demonstrated, the underlying mechanisms are likely multifactorial.

An often-proposed explanation is that previous childbirth provides women with practical knowledge of labor and contributes to more realistic expectations, whereas first-time mothers may encounter greater uncertainty and lower confidence in their ability to manage labor. This interpretation is consistent with psychological models of childbirth that emphasize childbirth self-efficacy, perceived control, and emotional preparedness.

Interestingly, the own capacity domain received the lowest score among all four CEQ2 dimensions in the study. Similar findings have been reported in several international validation studies of the CEQ2, although Slovak women appeared to report slightly lower levels of confidence and control than women from Northern European countries^10,16-18^. This observation may reflect differences in maternity care models rather than differences in obstetric outcomes alone. Although many European healthcare systems have progressively implemented principles of woman-centered maternity care, including shared decision-making, continuity of midwifery care, and active maternal participation during labor, maternity care in Slovakia continues to be predominantly hospital-based and medically oriented. Under such circumstances, women may have fewer opportunities to exercise autonomy during childbirth, potentially reducing their confidence and perceived control despite receiving clinically appropriate care, a concern supported by recent Slovak data indicating that women’s perceived autonomy in decision-making during childbirth is closely related to their birth satisfaction^22^. This interpretation, however, remains hypothetical and could not be directly examined in the present study.

### Mode of birth and women’s perception of safety

Another important finding was the association between operative vaginal delivery and a less positive childbirth experience, particularly in the perceived safety domain. On the contrary, the cesarean section was not independently associated with childbirth experience after adjustment for parity and other demographic and obstetric characteristics. This finding suggests that the negative association between operative birth and childbirth experience may be more pronounced after operative vaginal delivery than after cesarean section in this study population. Previous studies have also identified mode of birth as an important factor associated with childbirth experience, although the strength and consistency of associations with specific modes of birth vary between studies^11-13^.

The relationship between operative birth and less positive childbirth experience is unlikely to be explained solely by the intervention itself. Rather, operative birth frequently occurs in situations characterized by prolonged labor, fetal compromise, unexpected complications, or urgent clinical decision-making^24^. Such circumstances may reduce women’s opportunities to participate in decisions, diminish their sense of control, and increase feelings of uncertainty and vulnerability. In particular, the association between mode of birth and childbirth experience remained present after adjustment for demographic characteristics, which is consistent with previous research suggesting that subjective childbirth experience is related not only to what happens during labor but also to how women experience communication, involvement and emotional support throughout that process^6,7,10,25,26^. However, given the cross-sectional design, these interpretations should be regarded as hypotheses rather than as established causal pathways.

### Professional support as a potential protective factor

Professional support achieved moderately high scores in the present study. Although the overall regression model for this domain reached statistical significance, none of the demographic or obstetric variables examined was associated with the women’s ratings after adjustment. This finding suggests that perceptions of professional support may be influenced by other aspects of maternity care, such as communication, respectful treatment, emotional support, continuity of care, or individual expectations, rather than by maternal or obstetric characteristics alone.

Professional support is consistently recognized in the literature as one of the key modifiable factors associated with maternal satisfaction. Previous studies have shown that women who perceive healthcare professionals as respectful, empathetic, and responsive report higher childbirth satisfaction, regardless of obstetric interventions or clinical outcomes^6,7,10,25,26^. Effective communication, continuous emotional support, and participation in decision-making have all been identified as important components of high-quality maternity care. The absence of significant associations between demographic or obstetric characteristics and the professional support domain after adjustment, suggests that women’s evaluations of professional care may depend less on clinical circumstances than on the quality of interpersonal interactions with healthcare professionals. This finding reinforces the concept that respectful maternity care extends beyond technical competence and encompasses empathy, clear communication, emotional support, and continuity of care throughout labor and childbirth.

### Participation and the transition towards woman-centered maternity care

Participation received the highest score among all CEQ2 domains, suggesting that most women perceived themselves as involved in decisions related to their childbirth. However, multiple regression analysis showed that none of the demographic or obstetric characteristics examined was associated with the participation ratings of the women. This finding suggests that perceived involvement in decision-making may depend more on organizational and interpersonal aspects of maternity care than on maternal characteristics or birth outcomes.

Participation and shared decision-making constitute fundamental principles of contemporary woman-centered maternity care and have consistently been associated with more positive childbirth experiences^2,10,17,18,22^. Similar findings have been reported in several European countries, where increasing emphasis has been placed on respecting women’s autonomy and promoting collaborative relationships between women and healthcare professionals. Furthermore, relatively high participation scores may also reflect cultural expectations regarding childbirth care, limited familiarity with participatory models of maternity care, prevailing institutional norms, or a general sense of satisfaction and relief reported shortly after birth. Therefore, these considerations should be regarded as possible explanations rather than conclusions derived from the present data, and more research would be needed combining patient-reported measures with observational data to examine the actual extent of shared decision making in routine practice.

However, these findings should be interpreted with caution because the participation domain reflects subjective perceptions of women rather than objective measures of participation in clinical decision-making. Consequently, relatively high participation scores do not necessarily indicate consistent implementation of shared decision-making in all maternity settings.

### Strengths and limitations

One of the main strengths of this study is the use of a Slovak translation of the internationally validated Childbirth Experience Questionnaire 2 (CEQ2), which enables the comparison of women’s childbirth experiences with findings from other countries and contributes to the growing body of international evidence on woman-centered maternity care. To our knowledge, this is among the first studies to apply the CEQ2 in a Slovak maternity care setting while simultaneously examining demographic and obstetric predictors of both the overall childbirth experience and its individual domains using multivariate regression analysis. This approach provides a more comprehensive understanding of the factors shaping women’s childbirth experiences than descriptive analyses alone. Another strength is the inclusion of women representing different modes of birth and parity, allowing evaluation of key obstetric determinants within the same study population. Furthermore, data were collected during the early postpartum period, when women’s recollections of labor and birth remained recent, thus reducing the likelihood of long-term recall bias.

Several limitations should also be considered when interpreting the findings. First, the cross-sectional design does not allow causal relationships to be established; all reported associations should be interpreted as correlational. Second, childbirth experience was evaluated using a self-reported questionnaire, and the responses may consequently be subject to reporting bias and social desirability bias, particularly given that the data were collected shortly after birth in the hospital setting. Third, the study was carried out in a single regional maternity hospital, which introduces potential selection bias and may limit the generalizability of the results to other healthcare settings within Slovakia or internationally. Fourth, no *a priori* sample size calculation was performed, as all eligible women identified during the predefined recruitment period were included. Therefore, the relatively modest sample size may have reduced statistical power to detect smaller associations.

Childbirth experience was evaluated shortly after birth; consequently, the responses of the women may have been influenced by their immediate emotional and physical condition^27^. Although regression models explained a meaningful proportion of variance in several CEQ2 domains, childbirth experience is a complex phenomenon influenced by numerous psychological, social, and organizational factors beyond the scope of the present study, such as previous childbirth expectations, labor pain, birth companion, labor induction, obstetric analgesia, continuity of care, socioeconomic status, and partner support.

Fifth, although overall CEQ2 demonstrated acceptable internal consistency (Cronbach’s α=0.760), lower reliability coefficients were observed for the professional support (α=0.564) and participation (α=0.446) domains. This may partly reflect the relatively small number of items within these domains and the modest sample size of the present study. In addition, although multicollinearity was assessed using variance inflation factors and tolerance statistics and no evidence of problematic multicollinearity was identified, other regression assumptions, including residual normality and homoscedasticity, were not formally evaluated. As these unmeasured factors may be related to both the examined variables and childbirth experience, residual confounding cannot be excluded.

### Clinical implications

Although the cross-sectional design of this study does not allow causal conclusions to be drawn, the present findings, considered together with the existing literature, may help identify areas that deserve attention in maternity care. The observed associations suggest that nulliparous women may represent a relevant target group for antenatal education aimed at supporting childbirth confidence and realistic expectations, and that respectful communication, timely information and shared decision-making may be particularly important for women undergoing operative birth. These directions are consistent with international recommendations on respectful, woman-centered maternity care^2-4,25,26,28^; however, whether such interventions improve childbirth experience in the Slovak context would need to be evaluated in future intervention studies.

### Future research

Future research should include larger multi-center studies involving maternity hospitals representing different geographical regions and models of maternity care to improve the generalizability of the findings. Longitudinal studies that follow women beyond the early postpartum period would provide valuable insight into the stability of childbirth experiences over time and their relationship with maternal psychological well-being, breastfeeding, mother-infant bonding, and future reproductive intentions.

Further research should also investigate organizational and psychosocial determinants that were not examined in the present study, including continuity of midwifery care, childbirth expectations, perceived autonomy, partner support, and socioeconomic characteristics. Comparative studies across Central and Eastern European countries would be particularly valuable for understanding how differences in maternity care organization influence women’s childbirth experiences and for identifying effective strategies to strengthen women-centered maternity care throughout the region.

## CONCLUSIONS

The present study provides preliminary evidence on women’s childbirth experiences in a regional maternity hospital in Slovakia using the Childbirth Experience Questionnaire 2 (CEQ2). Although women generally reported positive childbirth experiences, differences were observed between individual domains, with participation rated the most favorably and own capacity least favorably, indicating that clinical results alone do not fully account for how childbirth is perceived. Consistent with previous international studies, multiparity was associated with a more positive overall experience of childbirth and higher scores in the domains own capacity and perceived safety, while operative vaginal delivery was associated with a less positive overall experience and lower perceived safety. In contrast, no demographic or obstetric factors were independently associated with the professional support or participation domains, suggesting that women’s perceptions of these care aspects may be related to the interpersonal and organizational characteristics of maternity care rather than to maternal or obstetric characteristics alone. Given the cross-sectional design, the single-center setting, and the modest sample size (n=103), these findings should be interpreted as exploratory associations rather than causal relationships. Larger, multi-center, and longitudinal studies are needed, together with more robust analyses of the factors associated with each CEQ2 domain, to confirm the observed associations and to further examine women’s childbirth experiences in Central European maternity care settings.

## Data Availability

The data supporting this research are available from the authors on reasonable request.
